# Hashimoto Thyroiditis, but Not Hypothyroidism Due to Other Causes, Is Associated with Lower Phase Angle Score in Adult Women: A Pilot Cross-Sectional Study

**DOI:** 10.3390/jcm12010056

**Published:** 2022-12-21

**Authors:** Ewelina Polak-Szczybyło, Agnieszka Ewa Stępień, Magdalena Zielińska, Mariusz Dąbrowski

**Affiliations:** 1 Department of Dietetics, Institute of Health Sciences, University of Rzeszow, College of Medical Sciences, 35-959 Rzeszów, Poland; 2 Institute of Medical Sciences, University of Rzeszow, College of Medical Sciences, 35-959 Rzeszów, Poland

**Keywords:** Hashimoto’s thyroiditis, hypothyroidism, inflammation, bioimpedance analysis, phase angle

## Abstract

Purpose: In recent years, Hashimoto’s thyroiditis (HT) has become one of the commonest autoimmune diseases. Its clinical symptoms include systemic manifestations related mainly, but not solely, to thyroid hormone deficiency. The bioimpedance phase angle (PhA) is a reliable indicator of nutritional as well as cellular health status. PhA is decreased not only in malnutrition, but also in many inflammatory diseases. The aim of this study was to assess the relationship between HT and PhA score. Methods: In this cross-sectional study, we compared the anthropometric, laboratory and body composition parameters of 49 women with HT and 98 propensity-score-matched women without thyroid disease. Results: Females with HT had significantly lower PhA scores (5.78 ± 0.43) compared to women without a thyroid disorder (5.98 ± 0.49, *p* = 0.017). Regarding TSH levels, although they were within the normal range in all study participants, in women with HT, they were slightly but significantly higher. Women with HT treated with L-thyroxin had significantly higher PhA compared to their non-treated counterparts. No differences between women with non-HT hypothyroidism and the control group were found. Conclusions: Decreased PhA scores in patients with HT may indicate that the inflammatory process in this autoimmune disease has an influence on cellular health and on the function of the entire body. Such an association was not found in women with non-HT hypothyroidism.

## 1. Introduction

Hashimoto’s thyroiditis (HT) is an autoimmune inflammatory disease associated with the presence of autoantibodies against thyroglobulin (TG) and thyroid peroxidase (TPO), and with lymphocytic infiltration of the thyroid gland [1,2,3,4]. HT has become one of the most common thyroid pathologies, with its incidence reaching 0.3 to 1.5 new cases per 1000 people per year [2]. Its prevalence is 10 times higher among women compared to men, and in more than 10% of females, thyroid autoantibodies are present [3]. The exact mechanisms of HT development have not been fully elucidated; however, an interplay between genetic susceptibility, environmental triggers (including diet) and epigenetic factors seems to play the most important role. Its clinical symptoms are not limited to the thyroid gland, but also include systemic manifestation related mainly, but not only, to thyroid hormone deficiency [1,2,3,4].

The electrical properties of the human body have been known since the late 19th century. A pioneer in this field was Hermann, who was the first to describe this phenomenon in 1871 [5]. On this basis, bioelectrical impedance analysis (BIA) has been developed and the principles of this method were established in the 1970s [6]. BIA has become a common, non-invasive and non-expensive method of examining body composition in health and disease, in people within different age ranges, and in different populations [7,8,9,10]. It is one of the most popular methods for assessing nutritional status. Its test–retest reliability coefficient, with correct measurement in the four-electrode system, is 99%. The advantages of BIA include its speed and repeatability. The method is safe and enables numerous parameters of body composition to be obtained, including fat and lean mass, the distribution of body water in intracellular and extracellular compartments, body cell mass (BCM) and phase angle (PhA). The assessment of PhA is based on the measurement of impedance (Z), i.e., the electrical resistance of the tissues, during the flow of low currents (≤1 mA) [7,8,9,10,11]. The impedance consists of reactance (Xc) and resistance (R). Resistance depends on the water and electrolyte content in tissues, while reactance reflects the cell membrane capacitance (i.e., its ability to save electrical charges by the non-conducting object), reflecting its integrity, function and biochemical structure; it is responsible for approximately 10% of impedance [12,13]. PhA is calculated using the formula:

$$\mathrm{PhA}=\frac{R}{\mathrm{Xc}}\times \frac{180{}^{\circ}}{\pi }$$

The phase angle not only reflects the integrity of cell membranes, but also indicates the shift of water from the intracellular to the extracellular space. It also expresses the ratio of extracellular mass (ECM) to total BCM [7,13,14,15]. The European Society of Clinical Nutrition and Metabolism (ESPEN) recognizes the phase angle as a reliable prognostic indicator and measure of nutritional status [16,17]. The PhA score is considered a prognostic tool in cancer patients’ survival [18]. PhA is decreased in many chronic diseases such as liver cirrhosis [15,19], chronic kidney disease [20], inflammatory bowel diseases [21], psoriasis [22], diabetes [23,24] and heart failure [25].

The aim of this study was to assess body composition—including the phase angle score, TSH, fasting insulin and plasma glucose levels, via calculation of the Homeostasis Model Assessment of Insulin Resistance (HOMA-IR) index [26]—in a group of women suffering from HT compared to females without thyroid disease.

## 2. Materials and Methods

### 2.1. Study Subjects

**Figure 1:**
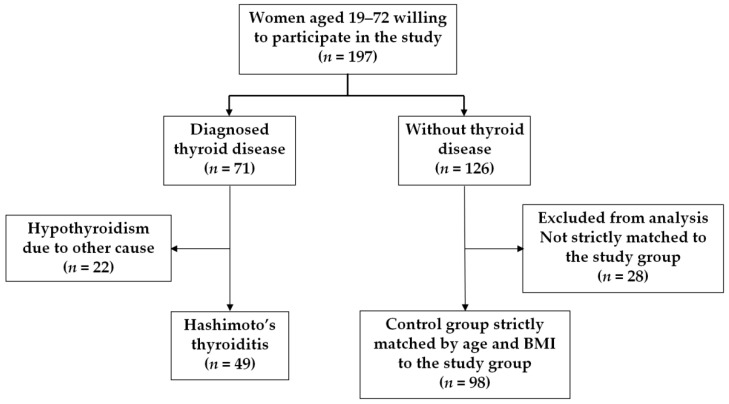
Flow chart of study participant selection.

In this cross-sectional study, 197 women aged 19–72 and treated in a dietary clinic were included. In this group, 71 females suffered from thyroid disease. Of these women, 49 were diagnosed with HT and 22 had hypothyroidism due to other causes. All of them were diagnosed and treated by endocrinologists in endocrine outpatient clinics. The remaining 126 females did not suffer from thyroid disease. The study group consisted of 49 women with autoimmunological thyroiditis (34 treated with L-thyroxin). The control group consisted of females without thyroid disease, strictly matched to the study group by age and BMI (both of which strongly correlate to PhA score) [27]. We used a propensity-score-matching method in a 2:1 ratio. The age range was identical in both groups—21–53 years (4 younger and 11 older women without thyroid disease were excluded from analysis before the propensity-score-matching procedure). A flow chart of study participant selection is presented in Figure 1. In all study participants, TSH levels were within the normal range. The exclusion criteria included: pregnancy, epilepsy, an implanted pacemaker, prostheses and an inability to maintain an upright body posture. The study participants were advised to fast for at least 10 h, and to not consume alcohol or diuretics and to avoid intense physical exercise for 24 h before the study procedures.

### 2.2. Anthropometric Measurements and Bioelectrical Impedance Analysis

Prior to the body composition assessment, each person’s height was measured twice using a Tanita HR-200 measuring rod (Tanita, Tokyo, Japan), and the result was rounded to the nearest 0.5 cm. Body composition was assessed with the use of the bioimpedance analyzer BIA MC-780 P MA (Tanita, Tokyo, Japan), with variable frequencies of: 5 kHz, 50 kHz and 250 kHz. It has four pairs of electrodes, which enables segmental body composition analysis. Its accuracy in measuring body weight is ±100 g. The device meets the non-automatic weighing instruments (NAWI) class III standards according to the directive 2014/31/EU. The measured parameters included fat mass (FM), fat-free mass (FFM), muscle mass (MM), total body water (TBW), intracellular (ICW) and extracellular water (ECW) (in kg and %), visceral fat index, impedance Z and phase angle (PhA). The obtained results were analyzed with the use of GMON consumer software (Tanita, Tokyo, Japan).

### 2.3. Laboratory Tests

Fasting blood samples were collected between 7:00 and 9:00 after at least 10 h of fasting, in a certified, commercial diagnostic laboratory. The levels of TSH, insulin and plasma glucose were determined, then, the HOMA-IR index was calculated using the following formula [26]:

$$\mathrm{HOMA}-\mathrm{IR}=\frac{\mathrm{insulin}\;\left(\mathrm{\mu IU}/\mathrm{mL}\right)\times \;\mathrm{glucose}\;\left(\mathrm{mmol}/L\right)}{22.5}$$

### 2.4. Statistical Analysis

Statistical analysis of the data was performed using SigmaPlot for Windows, version 12.5 (Systat Software Inc., San Jose, CA, USA). The continuous variables are presented as mean and standard deviation (SD), while the categorical variables are presented as numbers and percentage (in parentheses). The normality of the data distribution was checked using the Shapiro–Wilk test. Differences between the groups were analyzed using an unpaired two-tailed Student’s *t*-test or via a Mann–Whitney rank sum test where appropriate. The categorical variables were compared using a χ^2^ test with the Yates continuity correction applied. We also analyzed the area under the curve (AUC) of the receiver operating characteristics (ROC) curve for the assessment of the association between PhA and the presence of thyroiditis. The linear correlation between the PhA score and the analyzed variables was assessed with the use of a Spearman Rank Order Correlation test. We assumed a *p* value of < 0.05 to be statistically significant.

## 3. Results

The demographic, anthropometric and laboratory data of the women from the study and control groups are presented in Table 1. The age and BMI were almost identical in both groups. The distribution of normal weight, overweight and different grades of obesity was also not significantly different. Fasting insulin and plasma glucose in the two groups, as well as the HOMA-IR values, were similar. We only found significantly higher TSH levels in the study group, despite the fact that all study participants had TSH values within normal range.

The body composition parameters, including the impedance Z and PhA values, are presented in Table 2. Impedance Z was significantly higher in the study group, while the PhA score, despite the relatively small difference, was found to be significantly lower. Although more women in the study group apparently had PhA values below the 10th percentile for age, gender and BMI, the difference did not reach the limit of statistical significance. The reference values for this analysis were taken from the study of Bosy-Westphal conducted in 214,732 German adults, because the Polish population can be assumed to be similar to the neighboring German population [27]. Other parameters of body composition were not significantly different (only the percentage of ICW tended to be lower in the HT group).

**Figure 2:**
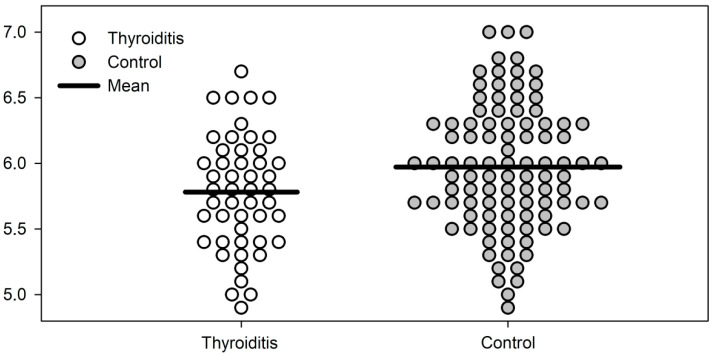
Dot density plot of phase angle score in the Hashimoto’s thyroiditis and control groups.

**Figure 3:**
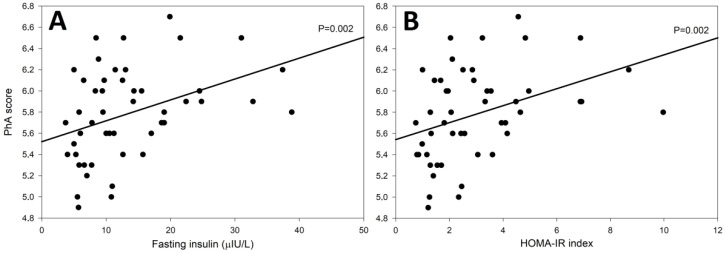
Linear correlation between phase angle (PhA) score and fasting insulin level (**A**), and with HOMA-IR value (**B**) in women with Hashimoto’s thyroiditis: scatter plot and regression lines.

The distribution of PhA scores in both groups is presented in Figure 2. The ROC curve analysis revealed a significant association between PhA score and HT (AUC = 0.617, *p* = 0.021). Moreover, we found a strong positive linear correlation between fasting insulin levels and HOMA-IR index and the PhA score (R = 0.439, *p* = 0.002 in both cases) (Figure 3).

Due to the fact that the PhA score correlates with BMI, we analyzed differences between the study and control groups, divided into four subgroups according to BMI category. Compared to the control group, women with HT apparently had lower PhA scores in a subgroup of normal-weight and obese subjects. This difference appeared to be significant in the highest BMI category (Table 3).

Females treated with L-thyroxin (*n* = 34), compared to non-treated women (*n* = 15), had significantly higher PhA scores (5.87 ± 0.42 vs. 5.57 ± 0.40, *p* = 0.023), despite TSH levels not being significantly different between the groups (2.31 ± 1.33 μIU/L vs. 2.15 ± 1.39 μIU/L, respectively). Females with PhA scores below the 10th percentile tended to have lower fasting insulin levels (8.42 ± 3.42 μIU/L vs. 14.30 ± 9.12 μIU/L, *p* = 0.065) and a lower HOMA-IR index (1.55 ± 1.27 vs. 2.71 ± 1.71, respectively, *p* = 0.063).

To check that a lower PhA score is associated with HT and not with hypothyroidism due to other causes, we compared 22 women with hypothyroidism that was not due to HT, and all 126 women without thyroid disease (age and BMI were not significantly different between the groups, and thus, we did not perform propensity-score matching). Apart from fasting plasma glucose, which was significantly lower in women without thyroid disease, no significant differences were found. The results are presented in Table 4 and Table 5. Because all parameters of body composition were insignificantly different between these 2 groups, they are omitted in Table 5.

## 4. Discussion

The relationship between the phase angle score and HT has not been studied yet. Our study was the first to analyze such a relationship. We revealed that women with HT had a significantly lower PhA score compared to the control group. Additionally, significantly more women in the study group (18.4%) had a phase angle score below the 10th percentile for age, BMI and gender, compared to females without thyroid disease (5.1%).

HT is an autoimmune inflammatory disease and is the commonest autoimmune thyroid disorder [2]. It is characterized by the lymphocytic infiltration of thyroid parenchyma, increased volume of the thyroid gland, and the presence of antibodies specific to thyroid antigens in the blood; however, clinically overt hypothyroidism develops in only 20-30% of patients [2,3]. It has been hypothesized that the inflammatory process is initiated and sustained as a result of the altered function of a subpopulation of CD8+ suppressor T cells, the decreased activity of regulatory lymphocytes (Treg) and the increased activity of T-helper lymphocytes (Th). The latter stimulate B cells and cause their transformation into plasma cells producing antibodies against thyroid antigens, leading to thyroiditis [3,28]. Additionally, an excessive number of DNA fragments following cell death are released from thyroid tissue. They can alter the microRNA profile, which results in initiation and perpetuation of the autoimmune process [4]. All these phenomena are associated with the increased release of pro-inflammatory cytokines (IFN-γ, IL-12, TNF-α, IL-1β, IL-2, IL-17, IL-22 and IL-18) and with the increased expression of multiple inflammasome components (NLRP1, NLRP3, NLRC4, AIM2, ASC and caspase-1), which may enhance the autoimmune response [1,3,28]. Additionally, a growing body of evidence indicates that HT is associated with an increased risk of thyroid cancer [3,29]. Systemic symptoms of HT are mainly associated with less or more pronounced hypothyroidism [1,2,3,4]. However, the presence of thyroid autoantibodies was also associated with inflammatory arthropathy, which has been recently described in patients with HT [30,31]. Moreover, in patients with HT, neuropsychiatric symptoms constituting a picture of so-called Hashimoto’s encephalitis were described, irrespective of auto-antibody titer and thyroid function [32]. Thyroid autoimmunity in pregnant women was associated with miscarriage and preterm delivery, despite clinical euthyroidism [33]. In a recently published paper, HT accompanying type 1 diabetes in young women (mean age <30), adequately controlled with levothyroxine, was associated with significantly greater carotid intima-media thickness (which is considered a marker of subclinical atherosclerosis) compared with patients with type 1 diabetes, but without HT [34]. These data indicate that autoimmune thyroiditis may be associated with inflammatory processes that are not limited to the thyroid gland only, and not necessarily related to thyroid function.

The PhA score has been found to be significantly decreased in many autoimmune as well as inflammatory diseases. In pediatric patients with newly diagnosed type 1 diabetes mellitus, which is characterized by severe insulin deficiency, PhA was significantly reduced compared to their healthy counterparts [24]. Interestingly, in our study, a correlation between insulin level and PhA score was also found. This may be a potential indicator of an early phase of autoimmune destruction of the pancreatic beta cells that can lead to the development of type 1 diabetes, as these two autoimmune diseases often accompany each other. Unfortunately, the presence of islet autoantibodies has not been assessed in HT patients. On the other hand, in another study conducted in children and adolescents with celiac disease, which is also an autoimmune disorder, the PhA score was not significantly different in the study group compared to healthy controls, despite significant differences in body composition [35]. Other disorders that significantly involve the immune system are inflammatory bowel diseases (IBD), such as Crohn’s disease (CD) and ulcerative colitis (UC). Studies conducted in patients under 18 years of age revealed that IBDs are associated with reduced PhA scores in comparison with the control groups, which was more pronounced in CD [21,36,37]. A comparative analysis of nutritional status and body composition in adult patients with CD and UC showed that patients with CD presented signs of malnutrition, and they had increased inflammatory activity and a significantly lower phase angle [38]. In IBD patients, both hand-grip strength and PhA were indicators of nutritional status and disease severity [39]. Immunosuppressive treatment resulted in an increase in PhA scores and FFM in patients with CD [40,41]. A common autoimmune disease that affects the functioning of the entire body is rheumatoid arthritis (RA). In this disorder, it was found that regardless of the BMI value, people with an exacerbation of RA had lower PhA values [42]. In patients with psoriasis, after the implementation of anti-IL12/23 therapy, the relief of psoriasis symptoms and a significant increase in body cell mass and phase angle scores was found [43]. Obesity is also accompanied by low-grade chronic inflammation, which occurs as a result of adipocyte hypoxia and is characterized by the accumulation of inflammatory cells and mediators, resulting in cell damage and/or death [44]. Changes in the phase angle score have also been correlated with the intensity of oxidative stress and with the level of inflammatory markers in the elderly [7,45,46]. In a study by Chen et al., PhA was significantly lower in overweight and obese subjects with non-alcoholic fatty liver disease (NAFLD) compared to the controls; however, in a subgroup analysis, it was found to be significant only in overweight, but not obese patients [47].

Obviously, our study is not free from limitations. The first and the most important one is the relatively small group of study participants. Because the statistical power of several tests was below the desired power of 0.800, the possibility of a random effect of some of the obtained results cannot be fully excluded. Moreover, it did not allow us to find other significant differences between the study and control groups (e.g., ICW tended to be lower in the HT group, which indicates a shift of water from intracellular to extracellular space, but the difference appeared to be insignificant). We also did not analyze other potentially important variables, e.g., thyroid antibody titers or CRP and pro-inflammatory cytokine level, because we did not have access to these data. Another limitation is the cross-sectional design of our study, which did not allow us to observe changes in the PhA score and other analyzed variables over time. Nevertheless, our study presents some new data and interesting findings which may indicate directions for future research.

## 5. Conclusions

To conclude, our study provided preliminary insights into the link between HT and decreased PhA scores in women. Decreased PhA in HT can be considered an indicator of the inflammatory process, not limited solely to the thyroid gland, but also present in the entire body. Interestingly, women with HT who were treated with L-thyroxin had significantly higher PhA scores, irrespective of TSH level. This phenomenon may indicate an important role of thyroid hormone supplementation in the attenuation of inflammatory processes and the restoration of metabolic balance. Another interesting finding is the correlation between insulin concentration and PhA. Thus, in such patients, an assessment of the presence of autoantibodies against islet antigens may be justified. Obviously, further research and larger studies, expanded with analyses of at least the abovementioned variables, are needed; with all the consequences, intervention options and predictive capabilities of PhA assessment in women with this disease, this could confirm our findings and determine the exact mechanisms of the impact of autoimmune thyroiditis on the entire body’s functioning.

## Figures and Tables

**Table 1:** Demographic, anthropometric and laboratory data of the women from the study and control groups. Significant differences are emphasized in italics.

Parameter	Thyroiditis, *n* = 49 Mean ± SD *	Control, *n* = 98 Mean ± SD *	*p* Value
Age (years)	35.12 ± 7.82	35.12 ± 8.32	0.847
BMI (kg/m^2^)	30.16 ± 6.15	31.35 ± 5.65	0.131
Normal weight (BMI < 25.0 kg/m^2^), *n* (%)	9 (18.4)	11 (11.2)	0.322
Overweight (BMI 25.0–29.9 kg/m^2^), *n* (%)	18 (36.7)	29 (29.6)	
Obesity gr. I (BMI 30.0–34.9 kg/m^2^), *n* (%)	12 (24.5)	37 (37.8)	
Obesity gr. II/III (BMI ≥ 35.0 kg/m^2^), *n* (%)	10 (20.4)	21 (21.4)	
*TSH (μIU/L)*	*2.26 ± 1.33*	*1.79 ± 0.87*	*0.035*
Insulin (μIU/L)	13.22 ± 8.65	12.80 ± 7.04	0.662
Glucose (mg/dL)	90.41 ± 7.49	89.18 ± 8.33	0.387
HOMA-IR	3.00 ± 2.07	2.86 ± 1.71	0.823

* SD—standard deviation.

**Table 2:** Body composition parameters in the study and control groups. Significant differences are emphasized in italics.

Parameter	Thyroiditis, *n* = 49 Mean ± SD *	Control, *n* = 98 Mean ± SD *	*p* Value
Fat mass (kg)	31.48 ± 11.47	29.27 ± 8.64	0.936
Fat mass (%)	36.56 ± 6.84	34.92 ± 5.57	0.418
Fat-free mass (kg)	52.05 ± 5.82	52.85 ± 6.36	0.155
Fat-free mass (%)	63.44 ± 6.85	65.09 ± 5.57	0.410
Muscle mass (kg)	49.42 ± 5.52	50.18 ± 6.05	0.156
Muscle mass (%)	60.24 ± 6.50	61.79 ± 5.28	0.415
Total body water (kg)	37.29 ± 4.15	37.85 ± 4.49	0.166
Total body water (%)	45.45 ± 4.90	46.62 ± 4.01	0.383
Extracellular body water (kg)	16.72 ± 2.33	16.70 ± 2.21	0.305
Extracellular body water (%)	20.25 ± 1.37	20.49 ± 1.12	0.728
Intracellular body water (kg)	20.56 ± 1.99	21.15 ± 2.40	0.109
Intracellular body water (%)	25.20 ± 3.55	26.13 ± 2.92	0.420
Bone mass (kg)	2.63 ± 0.29	2.67 ± 0.32	0.162
Bone mass (%)	3.20 ± 0.35	3.29 ± 0.29	0.410
Visceral fat index	6.80 ± 3.23	6.36 ± 2.75	0.861
*Impedance (ohm)*	*636.20* ± *68.97*	*604.54* ± *66.29*	*0.008*
*Phase angle*	*5.78* ± *0.43*	*5.98* ± *0.49*	*0.017*
<10th percentile, *n* (%)	9 (18.4)	7 (7.1)	0.075
Normal, *n* (%)	40 (81.6)	91 (92.9)	

* SD—standard deviation.

**Table 3:** Phase angle score in study and control groups according to BMI categories. Significant differences are emphasized in italics.

Parameter	Thyroiditis, *n* = 49		Control, *n* = 98		*p* Value
	*n*	Phase Angle Mean ± SD *	*n*	Phase Angle Mean ± SD *	
Normal weight (BMI < 25.0 kg/m^2^)	9	5.49 ± 0.38	14	5.71 ± 0.40	0.191
Overweight (BMI 25.0–29.9 kg/m^2^)	18	5.91 ± 0.41	33	5.86 ± 0.57	0.728
Obesity gr. I (BMI 30.0–34.9 kg/m^2^)	12	5.78 ± 0.55	34	6.06 ± 0.42	0.080
*Obesity gr. II/III (BMI ≥ 35.0 kg/m^2^)*	*10*	*5.81 ± 0.26*	*17*	*6.29 ± 0.34*	*<0.001*

* SD—standard deviation.

**Table 4:** Demographic, anthropometric and laboratory data in the non-Hashimoto’s hypothyroidism group and in women without thyroid disease. Significant differences are emphasized in italics.

Parameter	Hypothyroidism, *n* = 22 Mean ± SD *	No Thyroid Disease, *n* = 126 Mean ± SD *	*p* Value
Age (years)	37.46 ± 11.36	37.06 ± 10.97	0.989
BMI (kg/m^2^)	32.69 ± 9.62	31.20 ± 5.71	0.686
Normal, *n* (%)	5 (22.7)	14 (11.1)	0.222
Overweight, *n* (%)	4 (18.2)	40 (31.7)	
Obesity gr. I, *n* (%)	6 (27.3)	45 (35.7)	
Obesity gr. II/III, *n* (%)	7 (31.8)	27 (21.4)	
TSH (μIU/L)	1.91 ± 1.05	1.77 ± 0.87	0.436
Insulin (μIU/L)	13.63 ± 14.72	12.73 ± 6.69	0.310
*Glucose (mg/dL)*	*94.90 ± 10.01*	*91.51 ± 14.89*	*0.017*
HOMA-IR	3.16 ± 3.33	2.94 ± 1.74	0.746

* SD—standard deviation.

**Table 5:** Impedance Z and phase angle in the non-Hashimoto’s hypothyroidism group and in women without thyroid disease.

Parameter	Hypothyroidism, *n* = 22 Mean ± SD *	No Thyroid Disease, *n* = 126 Mean ± SD *	*p* Value
Impedance (Ω)	600.05 ± 96.45	596.17 ± 69.39	0.821
Phase angle	5.99 ± 0.64	5.88 ± 0.51	0.375
<10th percentile, *n* (%)	0 (0)	13 (10.3)	0.242
Normal, *n* (%)	22 (100)	113 (89.7)	

* SD—standard deviation.

## Data Availability

Data are available at the University of Rzeszów Repository.
